# Psychometric properties of the EORTC QLQ-C30 in Uganda

**DOI:** 10.1186/s12955-021-01769-x

**Published:** 2021-04-23

**Authors:** Allen Naamala, Lars E. Eriksson, Jackson Orem, Gorrette K. Nalwadda, Zarina Nahar Kabir, Lena Wettergren

**Affiliations:** 1 Department of Nursing, College of Health Sciences, Makerere University, Kampala, Uganda; 2 Department of Medical Oncology, Uganda Cancer Institute, Kampala, Uganda; 3 Department of Learning, Informatics, Management and Ethics, Karolinska Institutet, 171 77 Stockholm, Sweden; 4 Medical Unit Infectious Diseases, Karolinska University Hospital, 141 86 Stockholm, Sweden; 5 School of Health Sciences, City, University London, London, EC1V 0HB UK; 6 Department of Neurobiology, Care Sciences and Society, Karolinska Institutet, Huddinge, Sweden; 7 Department of Women’s and Children’s Health, Karolinska Institutet, Stockholm, Sweden; 8 Department of Public Health and Caring Sciences, Uppsala University, Uppsala, Sweden

**Keywords:** Cancer, Cancer care, EORTC, Health-related quality of life, Quality of life, Sub-Saharan

## Abstract

**Background:**

Self-reported measures play a crucial role in research, clinical practice and health assessment. Instruments used to assess self-reported health-related quality of life (HRQoL) need validation to ensure that they measure what they are intended to, detect true changes over time and differentiate between subjects. A generic instrument measuring HRQoL adapted for use among people living with cancer in Uganda is lacking; therefore, this study aimed to evaluate the psychometric properties of the European Organisation for Research and Treatment of Cancer (EORTC) QLQ-C30 in patients with cancer in Uganda.

**Methods:**

Adult patients with various types of cancer (n = 385) cared for at the Uganda Cancer Institute answered the EORTC QLQ-C30 in Luganda or English language, the two most spoken languages in the country. The two language versions were evaluated with regard to data quality (floor and ceiling effects and missing responses), reliability (internal consistency) and validity (construct, known-group and criterion). Construct validity was examined through confirmatory factor analysis (CFA). Mean scores were compared between groups differing in disease stage to assess known-group validity. Criterion validity was examined according to associations between two QLQ-C30 subscales (Global quality of life and Physical function) and the Karnofsky Performance Scale (KPS).

**Results:**

Floor and ceiling effects were observed for several scales in the Luganda and English versions. All EORTC scales with the exception of Cognitive function (Luganda α = 0.66, English α = 0.50) had acceptable Cronbach’s alpha values (0.79–0.96). The CFA yielded good fit indices for both versions (RMSEA = 0.08, SRMR = 0.05 and CFI = 0.93). Known-group validity was demonstrated with statistically significant better HRQoL reported by patients with disease stages I–II compared to those in stages III–IV. Criterion validity was supported by positive correlations between KPS and the subscales Physical function (Luganda r = 0.75, English r = 0.76) and Global quality of life (Luganda r = 0.59, English r = 0.72).

**Conclusion:**

The Luganda and English versions of the EORTC QLQ-C30 appear to be valid and reliable measures and can be recommended for use in clinical research to assess HRQoL in adult Ugandans with cancer. However, the cognitive scale did not reach acceptable internal consistency and needs further evaluation.

## Background

Cancer is expected to increase worldwide by more than 85% by 2030 [1, 2]. Being diagnosed with cancer and undergoing its treatment may have a negative impact on patients’ health-related quality of life (HRQoL) and cause multiple concerns [3]. Measurements of HRQoL can be used for various purposes including to distinguish patients in need of actions and interventions at different periods during and after treatment [4].

A few studies have investigated HRQoL in persons suffering from cancer in East Africa using standardized measures. One such study conducted in Uganda assessed women with ovarian cancer using the abbreviated version of the World Health Organisation Quality of Life instrument and they found that patients scored poorly across domains [5]. In Tanzania and Kenya, quality of life have been assessed with the questionnaire developed by the European Organisation for Research and Treatment of Cancer (EORTC): the QLQ-C30 [6]. Masika and co-workers who assessed HRQoL with the EORTC QLQ-C30 in the capital of Tanzania, demonstrated low overall HRQoL in hospitalised patients, specifically with regard to financial situation and pain [7]. Their findings were supported by results from a cross-sectional study conducted in Kenya of approximately 150 women undergoing palliative treatment for cervical cancer [8].

The instrument established by the EORTC, the QLQ-C30, was originally developed in English to investigate HRQoL in patients participating in clinical trials [6]. The questionnaire is applicable in more than 70 countries worldwide including the major languages spoken in the Western world and Asia [9]. In Sub-Saharan Africa, the questionnaire is available in 9 languages: Afrikaans, Amharic, Ilokano, Kiswahili, Sepedi, Sotho, Xhosa, Yoruba and Zulu [9–12]. In Uganda no valid and reliable instrument for assessment of generic HRQoL in adults with cancer exists. Therefore, in this study, we set out to evaluate the psychometric properties of the Luganda and English versions of the EORTC QLQ-C30 in Uganda.

## Methods

The study employed a cross-sectional design.

### Study setting

The present study was conducted at UCI, which is the only public hospital providing specialised cancer services in Uganda. The services include screening, radiotherapy, chemotherapy, counselling, training programmes, rehabilitation and palliative care. Additionally, research and consultation services are offered to promote better cancer control and care in the country. The institute offers care for in- and outpatients and has a bed capacity of more than 100 inpatients organised into five clinical units: two units for children and three for adults (solid tumour centre, lymphoma treatment centre and an outpatient department). Annually, UCI receives over 60,000 new cancer cases of both men and women, of which cervical cancer and Kaposi sarcoma account for 19.7% and 13% of cases, respectively [13].

### Sample

Adult (≥ 18 years) patients with cancer, treated and cared for at UCI, speaking Luganda or English languages, with physical and mental ability (this was judged by the respective unit in-charges), were identified for possible participation in the study. A total of 482 patients were approached and screened for eligibility during a 4-week period (June–July 2019). Eligible patients (n = 407) were asked to answer the authorized Luganda [14] or English versions of EORTC QLQ-C30 (version 3). Data was collected by seven registered nurses who had been trained for the data collection procedure.

### The EORTC QLQ-C30

The EORTC QLQ-C30 version 3 consists of 30 items divided into nine multi-item subscales and six single items representing various aspects of HRQoL [6]. The multi-item scales include five functional scales (physical, role, emotional, cognitive and social), three symptom scales (fatigue, nausea/vomiting and pain) and a global health and quality-of-life scale (Global QoL). Additionally, six single items measure impact on symptoms of dyspnoea, insomnia, loss of appetite, constipation, diarrhoea and the perceived financial impact of the disease.

All items have four response alternatives that range from 1 to 4 (‘not at all’, ‘a little’, ‘quite a bit’ and ‘very much’), with the exception of the two items comprising the Global QoL scale, which have a score ranging from 1 (very poor) to 7 (excellent). According to the EORTC QLQ-C30 Scoring Manual, raw scores in QLQ-C30 are linearly transformed to 0–100 point scales [15]. For Global health status and the five functioning status, a score of 100 corresponds to a high HRQoL, whereas for financial difficulties and the eight symptoms, a score of 100 implies maximum difficulty or symptom burden [15, 16].

### Translation of the EORTC QLQ-C30 to Luganda language

For the purposes of this study, the instrument (version 3) was translated into the Luganda language and linguistically validated by our research group in accordance with the procedure developed by EORTC [17]. This included a forward–backward procedure, involving two forward and two backward translators. The steps (forward translation, reconciliation and backward translation with comments) were reviewed and approved by the Translational Unit of the EORTC Quality of Life Group before starting pilot testing. The preliminary Luganda version was pilot tested on 20 adult cancer patients, native Luganda speakers treated and cared for at the Uganda Cancer Institute (UCI) [14]. The translation report was reviewed by the EORTC translation coordinator before the Luganda version of the EORTC QLQ-C30 was authorised by the EORTC Quality of Life Group [9].

### Additional measures

#### The Karnofsky Performance Scale

All participants were assessed with the Karnofsky Performance Scale (KPS) index in addition to EORTC QLQ-C30. The KPS is an 11-point categorical scale ranging from 100 (normal functioning) to 0 (dead) in ten point increments [18]. The instrument may be used to compare functional abilities across populations as well as evaluating the effectiveness of different therapies [18, 19]. For data analysis patients were categorised into three groups [20], KPS of ≥ 80 reflected patients able to carry on with normal activity, those rated at 50–70 were unable to work but could take care of most personal needs while patients with KPS < 40 were unable to care for themselves and required care.

### Socio-demographic and clinical data

Demographic data were collected using study-specific items to assess sex, age, marital status, number of children and household members, level of education and employment status. Clinical information was collected through medical records and included cancer diagnosis and stage, date of diagnosis and type of received cancer treatment.

### Data analyses

Statistical analyses were performed in Stata version 14 [21]. Data were analysed by language version, Luganda and English. Analysis of data quality included distribution of scale scores (item means and standard deviations, floor and ceiling effects and missing item responses). The raw scores were used for psychometric evaluation, with the exception of floor and ceiling effects, which on the transformed scores [15]. Floor and ceiling effects were calculated and considered acceptable if they did not exceed 15% [22]. Cronbach’s alpha was used to assess internal consistency. For this measure, values ≥ 0.7 are considered ideal; however, some researchers consider values < 0.70 but close to 0.60 as satisfactory [23].

Construct validity was ascertained using confirmatory factor analysis (CFA), which was performed to determine the adequacy of the original EORTC QLQ-C30 on our data by language version. The maximum likelihood method was used in all analyses. When, as in our case, hypotheses exist, based on theory and previous analyses, CFA is more appropriate than exploratory factor analysis (EFA) [24]. Standardised coefficients of ≥ 0.4 were considered acceptable [25]. Normalized mean and covariance residuals were evaluated and found acceptable. Model fit was estimated by two absolute indices of overall model fit: root mean square error of approximation (RMSEA) and standardised root mean residual (SRMR). Additionally, one relative index of model fit was used: comparative fit index (CFI). The acceptable thresholds for these indices were defined as RMSEA = 0.05–0.08, SRMR < 0.08 and CFI > 0.90 according to Kline’s guidelines [26]. The degrees of freedom were reported, but not considered as an indicator of model fit owing to their restrictiveness of being sensitive to sample size [27].

Known-group validity was evaluated by examining the instrument’s capacity to discriminate between patients differing in disease stage (stages I–II vs. stages III–IV). Independent t-tests were calculated to investigate potential differences in the mean scores of the EORTC QLQ-C30 scales between the two groups. Effect sizes (ES) were calculated to indicate the clinical significance of possible differences in means, whereby ES = 0.2, 0.5–0.8 and > 0.8 were considered small, moderate and large difference, respectively [22].

Criterion validity was determined by calculating Pearson’s correlation coefficient for two of the EORTC QLQ-C30 subscales (Physical function and Global QoL) and the KPS [28]. It was hypothesised that the selected QLQ-C30 subscales would correlate positively to KPS with coefficients of large magnitude [28].

### Procedure

Data was collected by seven trained research assistants (nurses), who approached patients in the clinical units. Patients were identified through patient lists generated by the study coordinator at the wards and outpatient department. The lists were used to evenly distribute participants among data collectors with the help of unit in-charges as well as planned appointments.

Potential participants were given written and oral information in Luganda or English about the aim and procedures of the study. It was stressed that participation was voluntary and that non-participation would not affect care and treatment in any way. Informed consent was asked for, and those patients who could not provide consent in writing were asked to do so through thumb print. Due to the large number of patients seen at UCI who could not read or write, the questions were read to all patients. Additional demographic and clinical data were collected from patients’ clinical records at UCI.

## Results

Of the 482 approached patients, 75 were excluded due to the following: poor health (n = 23), ongoing staging investigations (n = 15), cognitive difficulties (n = 5) or language barrier (n = 32). Furthermore, 22 patients refused to participate, whereas 385 (66% women and 34% men aged 18–84 years) consented to participate and subsequently answered the EORTC QLQ-C30 in Luganda (n = 217) or English (n = 168) by interview, resulting in a response rate of 95%.

The sociodemographic characteristics of the sample are presented in Table 1. The clinical characteristics of the patients are presented in Table 2 and reveal that most participants (65%) were in-patients with cancers in stages III and IV. Furthermore, approximately 60% had received chemotherapy, and one quarter had received radiotherapy.

**Table 1:** Socio-demographic characteristics by language version, Luganda (n = 217) and English (n = 168)

	Total n (%)	Luganda n (%)	English n (%)	*χ*^2a^	Df	*P* value
**Sample size**	385 (100)	217 (100)	168 (100)			
**Sex**				1.10	1	0.294
Female	254 (66)	148 (68.2)	106 (63.1)			
Male	131 (34)	69 (31.8)	62 (36.9)			
**Marital status**				2.96	1	0.085
Married/cohabiting	217 (56.4)	114 (52.5)	103 (61.3)			
Widowed/single/divorced	168 (43.6)	103 (47.5)	65 (38.7)			
**Home region**				65.58	4	< 0.001
Northern	56 (14.5)	7 (3.2)	49 (29.2)			
Eastern	63 (16.4)	28 (12.9)	35 (20.8)			
Western	90 (23.4)	56 (25.8)	34 (20.2)			
Central	171 (44.4)	123 (56.7)	48 (28.6)			
Non-Ugandan	5 (1.3)	3 (1.4)	2 (1.2)			
**Education level**				46.83	3	< 0.001
Tertiary	65 (16.9)	15 (6.9)	50 (29.8)			
Secondary	123 (31.9)	63 (29)	60 (35.7)			
Primary	143 (37.1)	100 (46.1)	43 (25.6)			
None	54 (14)	39 (18)	15 (8.9)			
**Occupation**				17.58	3	0.003
Employed/farmer/business	323 (83.9)	181 (83.4)	142 (84.5)			
Student	14 (3.6)	5 (2.3)	9 (5.4)			
Housewife	8 (2.1)	4 (1.8)	4 (2.4)			
None	40 (10.4)	27 (12.4)	13 (7.7)			

Data collected at Uganda Cancer Institute 2019

^a^Differences in proportions tested with χ2 statistics

**Table 2:** Clinical characteristics of patients at Uganda Cancer Institute, Luganda language (N = (217) and English language (N = 168)

	Total n (%)	Luganda n (%)	English n (%)	*χ*^2b^	Df	*P* value
**Sample size**	385 (100)	217 (100)	168 (100)			
**Clinical setting**				0.39	1	0.533
In Patient	257 (66.8)	142 (65.4)	115 (68.5)			
Out Patient	128 (33.2)	75 (34.6)	53 (31.5)			
**Cancer type**				11.61	9	0.236
Cervical	92 (23.9)	60 (27.6)	32 (19.0)			
Breast	68 (17.7)	32 (14.7)	36 (21.4)			
KS	43 (11.2)	28 (12.9)	15 (8.9)			
Leukemia	26 (6.7)	14 (6.5)	12 (7.1)			
Prostate	22 (5.7)	12 (5.5)	10 (6.0)			
Oesophageal	20 (5.2)	8 (3.7)	12 (7.1)			
Lymphoma	18 (4.7)	7 (3.2)	11 (6.5)			
Lung	8 (2.1)	5 (2.3)	3 (1.8)			
Ovarian	8 (2.1)	4 (1.8)	4 (2.4)			
Other	80 (20.8)	47 (21.7)	33 (19.6)			
**Cancer Stage**				1.39	1	0.499
Early (I & II)	99 (25.7)	51 (23.5)	48 (28.6)			
Late (III & IV)	208 (54)	122 (56.2)	86 (51.2)			
Not reported^a^	78 (20.3)	44 (20.3)	34 (20.3)			
**Type of treatment**				0.02	1	0.813
Chemotherapy	235 (61)	130 (59.9)	105 (62.5)			
Radiotherapy	94 (24.4)	51 (23.5)	43 (25.6)			
Surgery	38 (9.9)	21 (9.7)	17 (10.1)			
Palliative	15 (3.9)	12 (5.5)	3 (1.8)			
No treatment yet	3 (< 1)	3 (< 1)	–			
**Grouped KPS**				0.41	2	0.813
≤ 40	58 (15.1)	34 (15.7)	24 (14.3)			
50–70	162(42.1)	93 (42.9)	69 (41.1)			
≥ 80	165 (42.9)	90 (41.5)	75 (44.6)			

DF, Degrees of freedom

^a^Missing data

^b^Differences in proportions tested with χ2 statistics

### Data quality

There were no missing item responses. Item means within subscales were roughly equivalent, and the standard deviations were close to 1 with the exception of the Global QoL scale. All response alternatives were used for all items. Floor effects in the Luganda version ranged between 5.5 and 53.0%, and ceiling effects ranged between 3.7 and 27.2% (Table 3). The corresponding floor effects for the English version ranged from 3.6 to 58.3%, and ceiling effects from 3.6 to 29.8%.

**Table 3:** Descriptive statistics for the subscales of EORTC QLQ-C30 by language version, Luganda (N = 217) and English (N = 168)

Subscales (no of items)	Mean^a^ (SD)	Range of item means (SD)	Floor/ceiling effect^b^, %	Cronbach’s α
**PF (5)**				
Luganda	2.47 (0.91)	1.74–3.10 (1.10–1.19)	8.8/9.2	0.86
English	2.31 (0.95)	1.61–2.90 (1.01–1.24)	7.7/14.9	0.88
**RF (2)**				
Luganda	3.00 (1.02)	2.89–3.10 (1.05–1.09)	35.5/11.5	0.91
English	2.77 (1.10)	2.77–2.77 (1.13–1.17)	28/18.5	0.90
**EF (4)**				
Luganda	2.29 (0.96)	2.16–2.38 (1.08–1.12)	9.7/13.8	0.89
English	2.26 (0.98)	2.15–2.52 (1.08–1.15)	8.9/21.4	0.91
**CF (2)**				
Luganda	2.17 (1.00)	2.12–2.21 (1.13–1.18)	11.1 /26.3	0.66
English	2.03 (0.91)	1.90–2.15 (1.08–1.15)	5.4/29.8	0.50
**SF (2)**				
Luganda	3.03 (1.03)	3.02–3.03(1.10–1.16)	41.0/9.7	0.79
English	3.08 (0.98)	3.01–3.15(1.01–1.12)	42.9/6.5	0.81
**FA (3)**				
Luganda	2.66 (0.96)	2.52–2.84 (1.04–1.08)	7.8/18.0	0.89
English	2.50 (0.98)	2.42–2.55(1.09–1.16)	14.3/9.5	0.85
**NV (2)**				
Luganda	1.82 (1.07)	1.72–1.93(1.07–1.16)	53.0/11.1	0.92
English	1.61 (0.92)	1.44–1.79 (0.88–1.08)	58.3/5.4	0.85
**PA (2)**				
Luganda	2.83 (0.99)	2.80–2.86(1.05–1.10)	9.2/27.2	0.83
English	2.70 (1.08)	2.70–2.70(1.10–1.18)	16.7/24.4	0.89
**Global QOL (2)**				
Luganda	4.02 (1.51)	4.02–4.02 (1.54–1.56)	5.5/3.7	0.94
English	3.93 (1.53)	3.89–3.98 (1.55–1.57)	3.6/3.6	0.96

PF; Physical function, RF; Role function, EF; Emotional function, CF; Cognitive function, SF; Social function, FA; Fatigue, NV; Nausea and vomiting, PA; Pain, Global QoL; Global quality-of-life

^a^Means and SDs calculated on raw scores; higher scores indicate better HRQoL and more symptoms

^b^Floor and ceiling effects calculated on transformed scores

### Reliability

Cronbach’s α ranged from 0.66 to 0.94 for the Luganda version and from 0.50 to 0.96 for the English version (Table 3). The Cognitive function scale had the lowest α (0.66, Luganda version; 0.50, English version).

### Construct validity

**Fig. 1:**
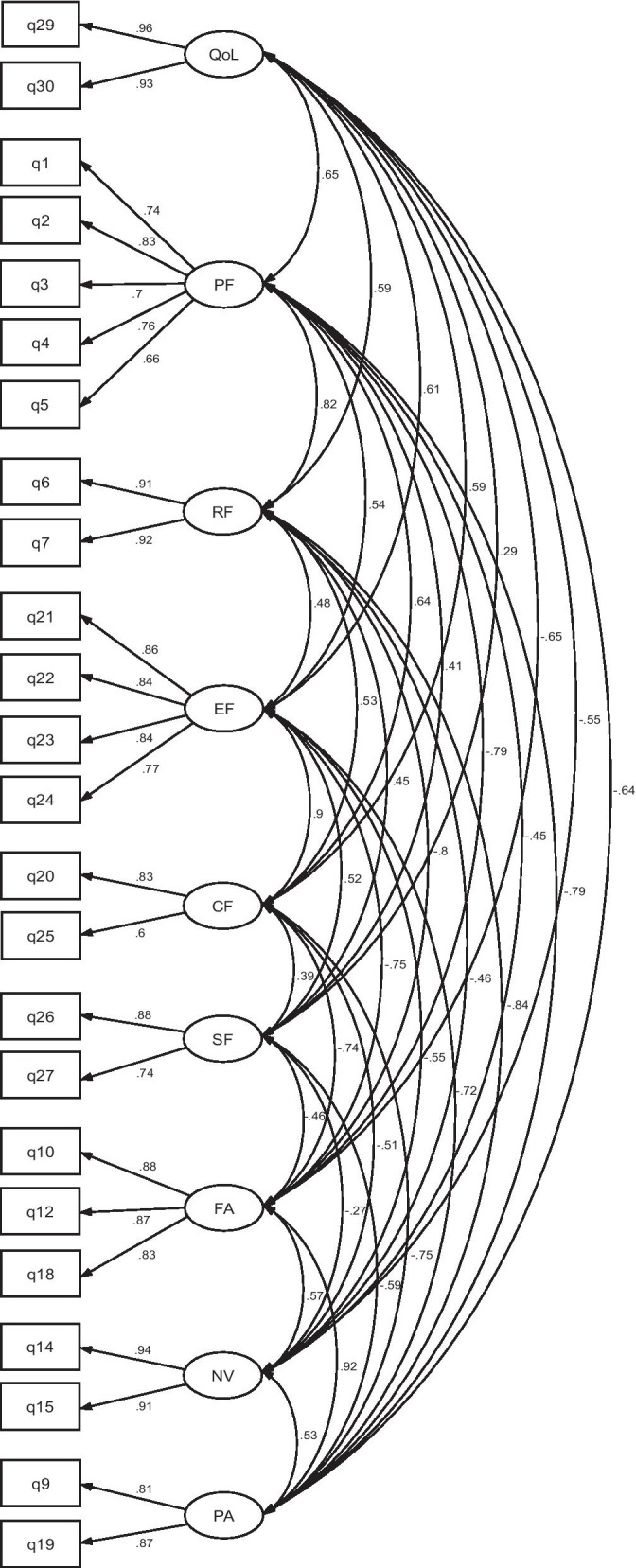
Model of confirmatory factor analysis (CFA) indicating standardized loadings on the factors of the Luganda version of the EORTC QLQ-C30 (n = 217)

**Fig. 2:**
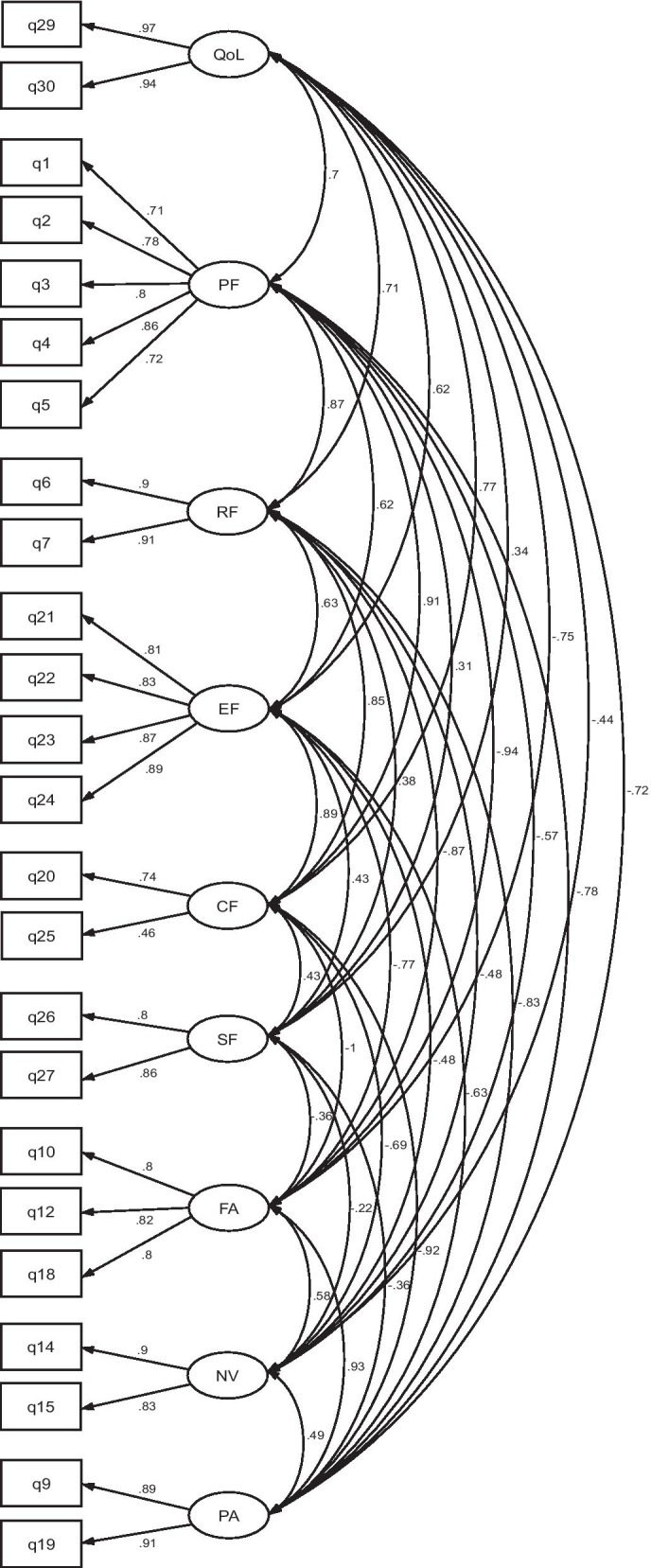
Model of confirmatory factor analysis (CFA) indicating standardized loadings on the factors of the English version of the EORTC QLQ-C30 (n = 168)

The CFA was performed separately on responses from the two versions: Luganda (n = 217) and English (n = 168), please see Figs. 1 and 2. Both versions provided statistically significant models: namely, Luganda *χ*^2^ = 490.07 (*p* < 0.001) and English version *χ*^2^ = 452.16 (*p* < 0.001) with standard factor loading estimates ranging from 0.59 to 0.96 for the Luganda version and from 0.46 to 0.97 for the English version. The fit statistics of the CFA conducted on the Luganda version provided an RMSEA of 0.08 (90% CI 0.07–0.09), an SRMR of 0.05 and a CFI of 0.93; the corresponding values for the English version were RMSEA = 0.08 (90% CI 0.07–0.09), SRMR = 0.05 and CFI = 0.93.

### Known-group validity

Mean values for early (I–II) and late (III–IV) stages of the disease (both language versions) are presented in Table 4. In two of the five functional subscales and the Global QoL scale, statistically significant differences (both languages) were detected between patients with late stage disease, who rated worse functioning compared to those in early stages. In the multi-item symptom scales the same pattern was seen with more symptoms among those in late stages, however, only statistically significant for the English versions. ES ranged between small and medium, with Physical function displaying the highest ES (Luganda 0.56, English 0.70). Some participants (n = 78) were still undergoing staging investigations and were not included in the analysis.

**Table 4:** Significant differences in self-reported HRQoL by disease stage, stage I-II (Luganda n = 51, English n = 48) compared to stage III-IV (Luganda n = 122, English n = 86)

QLQ-C30 subscales	Stage I-II Mean^a^ (SD)	Stage III-IV Mean^a^ (SD)	*P* value^b^	Effect size
**Global quality of life**				
Luganda	57.52 (26.94)	46.58 (24.64)	0.010	0.43
English	55.21 (23.67)	43.99 (26.12)	0.015	0.44
**Physical function**				
Luganda	60.39 (27.14)	44.01 (30.19)	< 0.001	0.56
English	68.19 (25.55)	46.51 (33.48)	< 0.001	0.70
**Role function**				
Luganda	43.14 (37.14)	26.37 (31.77)	0.003	0.50
English	49.65 (34.81)	36.24 (37.33)	0.043	0.37
**Cognitive function**				
Luganda	61.44 (33.91)	56.42(34.51)	0.382	0.15
English	69.10 (28.35)	61.82 (33.12)	0.202	0.23
**Emotional function**				
Luganda	55.56 (33.44)	52.80 (33.02)	0.619	0.08
English	62.33 (29.52)	55.23 (35.59)	0.243	0.21
**Social function**				
Luganda	21.57 (29.87)	30.33 (33.95)	0.111	− 0.27
English	25.69 (30.36)	30.23 (32.59)	0.430	− 0.14
**Fatigue**				
Luganda	52.29 (34.38)	61.57 (30.81)	0.083	− 0.29
English	42.13 (28.35)	56.85 (34.23)	0.013	− 0.46
**Nausea/vomiting**				
Luganda	20.59 (34.41)	32.24 (36.60)	0.054	− 0.32
English	13.19 (22.53)	24.42 (34.27)	0.044	− 0.37
**Pain**				
Luganda	58.17 (36.57)	66.80 (31.67)	0.121	− 0.26
English	47.92 (32.37)	62.60 (35.69)	0.020	− 0.42

Data collected at Uganda Cancer Institute 2019

ªMean scores ranges from 0 to 100, higher scores in functional scales reflect better HRQoL and higher scores in symptom scale more symptoms

^**b**^Differences tested by student’s unpaired *t*-test

### Criterion-based validity

Positive correlations were revealed between the KPS and the subscales of Physical function (Luganda version r = 0.75, English version r = 0.76) and Global QoL (Luganda version r = 0.59, English version r = 0.72), all tests were statistically significant (< 0.001) and the coefficients of large magnitude.

## Discussion

This study reported on the psychometric evaluation of the English and Luganda versions of the generic EORTC QLQ-C30 when used in cancer care in Uganda. Most importantly, the instrument demonstrated satisfactory validity in terms of construct and known-group validity as well as in criterion validity. However, even though the overall data quality was good with acceptable reliability (internal consistency) for all but one scale, considerable high floor and ceiling effects were detected for some scales, and this tendency merits some concern.

The study had a large sample of adult men and women cared for at UCI, including both in- and outpatients. While all hospitalized patients during the data collection period were approached indicating a census study, the recruitment of outpatients were not conducted in the same systematic way due to a large number of patients in the waiting areas. The participants of the study are still considered to be representative of the patients cared for at UCI which also is reflected by the vast majority speaking Luganda or English. Approximately 7% of the approached patients did speak neither Luganda nor English which is expected with more than 40 spoken languages in the country. Most Ugandans are peasant farmers who speak and conduct small scale businesses in local languages. No significant differences were found regarding gender, marital status and clinical characteristics with regard to language versions. This was expected considering the fact that cancer stage, type of treatment or status of KPS are independent of the language one speaks.

With regard to data quality, all response alternatives were used for all items (data not shown), suggesting that the response scale was sufficient. However, floor effects of approximately 30%–50% were observed for the Role function, Social function and Nausea/vomiting scales, with a similar pattern observed for both language versions. Additionally, ceiling effects exceeding 25% were observed in the Cognitive function and Pain scales for both language versions. Furthermore, ceiling effect of the English version of the Emotional function scale was 21%. This distribution may indicate that a large proportion of patients in an advanced disease stage experienced diminished functioning regarding role and social functioning due to fatigue and pain.

The high floor effects for the Nausea/vomiting scale could indicate poor ability to differentiate between no symptoms and mild symptoms; however, based on clinical experience, it might probably be partially a consequence of low prevalence of these symptoms in this patient population [16]. The high floor effects seen for the Nausea/vomiting scale was puzzling considering that approximately 60% of the patients were currently on chemotherapy and nausea and vomiting are well-known side effects of chemotherapy [29].

All scales but one had satisfactory reliability. The cognitive function scale displayed a Cronbach’s alpha of 0.50 for the English version and of 0.66 for the Luganda version. A low alpha coefficient for the cognitive scale has been reported in other studies in East Africa [7, 12, 30]. It has been argued that this may to some extent be due to the small number of items in the scale. One of the two items measuring cognitive function assesses the ability to concentrate on things (‘like reading a newspaper or watching television’), activities that may appear inappropriate for Ugandans with cancer since newspapers and televisions are not available to patients at UCI. Furthermore, the literature has shown that Ugandans have low reading literacy [31]. The other item in the scale asks about memory: ‘Have you had difficulty remembering things’. Thus, it is understandable that the two items may not be highly correlated and subsequently results in a low alpha value.

The CFA supported the notion that the EORTC QLQ-C30 measures the intended nine multi-item scales of HRQoL. Standardised factor loadings and the goodness of fit for outputs of the Luganda and English versions demonstrated satisfactory results, suggesting strong construct validity [23]. However, literature shows that the value of Cronbach’s alpha coefficients is highly influenced by the number of items in the scale, that alpha increases with the number of items in the domain. Six of the nine multi-item scales consist of only two items which is known to negatively impact on internal consistency of a scale [32], still satisfying results were seen for all scales with the exception of Cognitive function.

The instrument demonstrated known-group validity as the EORTC QLQ-C30 was able to successfully discriminate between early and late stages of cancer among participants, such that patients with advanced disease demonstrated poorer HRQoL than those with early stages of the disease. Furthermore, ES revealed that the detected statistically significant differences were clinically relevant. Furthermore, the physical function and Global QoL scales were both associated with the KPS, with large correlation coefficients indicating criterion validity [23].

### Strengths and limitations

This is the first study of HRQoL using the generic measure EORTC QLQ-C30 to be performed in Uganda. The study had a large representative sample of adult men and women cared for at UCI, including both in- and outpatients. The study was conducted using the most commonly used languages in the country. Still, a small proportion of patients could not speak and understand Luganda or English and were therefore excluded and if this group perceives the measure’s items in a different way is not known. Future studies carried out in Uganda are recommended to use educated interpreters to be able to include all Ugandan patients irrespective of spoken language. Furthermore, despite high efforts to reach patients during the data collection period we had difficulties to systematically include outpatients due to a large number of patients in the waiting rooms. Despite using trained interviewers instructed to include any outpatients available in the waiting area inclusion bias cannot be ruled out. The issue was discussed throughout the data collection and the interviewers were encouraged to reach out to all kind of patients (sexes, all ages, various health statuses, being accompanied or coming alone). Moreover, the cross-sectional design of the study prohibited any possibility to examine the instrument’s test–retest reliability.

## Conclusions

This first psychometric evaluation of the English and Luganda versions of the 30-item EORTC QLQ-C30 in a Ugandan context is promising. The instrument appears to be a valid and reliable measure, with the exception of the Cognitive function scale, in which we recommend that one item be rephrased to better suit a Ugandan context. Furthermore, the floor effects noted for Role and Social functioning, reflecting low function, as well as the ceiling effect for the Pain subscale (high pain) may be an obstacle when conducting comparative studies. Apart from caution in interpreting cognitive function, the instrument is recommended for clinical and epidemiological researchers to study the HRQoL of adult Ugandans with cancer.

## Data Availability

The datasets generated and/or analysed during the current study are available from the principal investigators of the study on reasonable request.

## Abbreviations

HRQoL: Health-related quality of life
PACT: Program of action for cancer therapy
RN: Registered nurse
QLQ-C30: Quality of Life Questionnaire (Version 3)
UCI: Uganda Cancer Institute
WHO: World Health Organisation
